# Supplementary material for: Lipocalin 2 mediates appetite suppression during pancreatic cancer cachexia
Source: Nat Commun. 2021 Apr 6;12:2057. doi: 10.1038/s41467-021-22361-3 (PMC8024334; doi:10.1038/s41467-021-22361-3)
Supplement: Supplementary file 1 — Supplementary Information [file 41467_2021_22361_MOESM1_ESM.pdf]

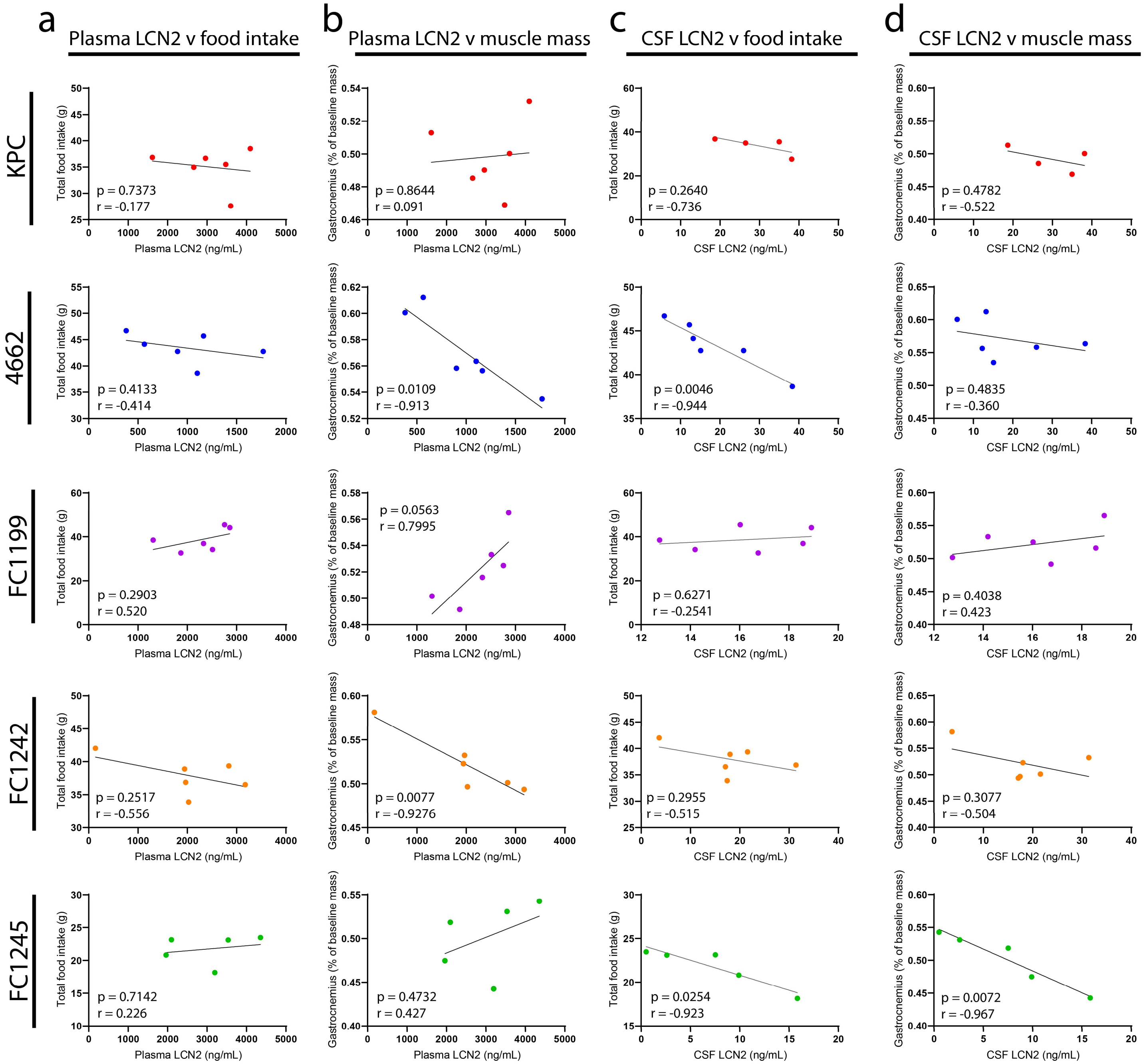

Supplementary Figure 1. Related to Figure 1K-N. Linear regression analysis between plasma LCN2 levels and (A) total food intake or (B) gastrocnemius mass for each individual pancreatic cancer cell line analyzed in Figure 1K-L. Linear regression analysis between CSF LCN2 levels and (C) total food intake or (D) gastrocnemius mass for each individual pancreatic cancer cell line analyzed in Figure 1M-N. Data were analyzed by simple linear regression and two-tailed correlation analyses. Sham operation controls = grey/black, KPC = red, FC4662 = blue, FC1199 = purple, FC1242 = orange, FC1245 = green.

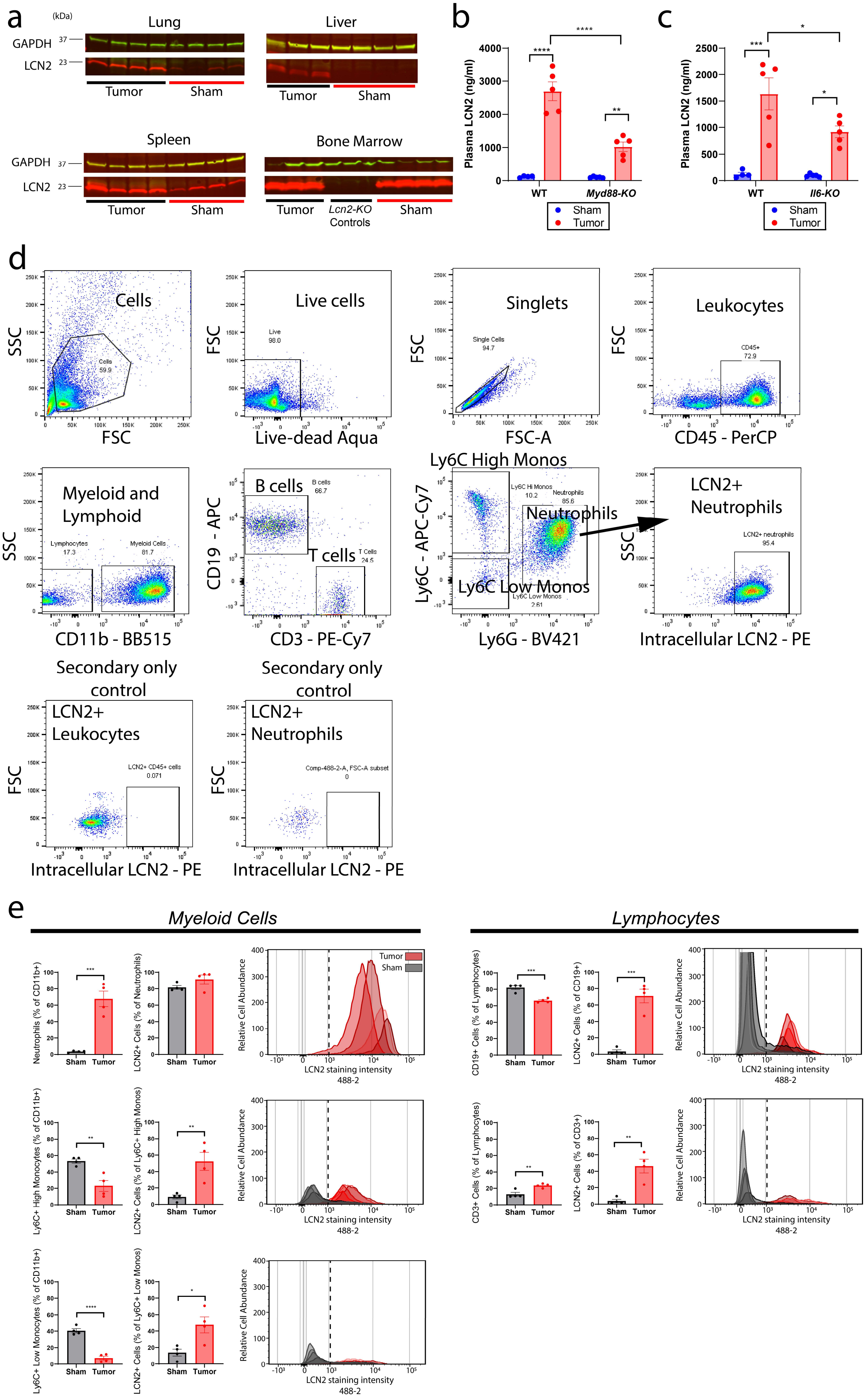

a
Lung
Liver
Spleen
Bone Marrow
GAPDH
LCN2
(kDa)
37
23
Tumor
Sham
Lcn2-KO Controls
b
Plasma LCN2 (ng/ml)
WT
Myd88-KO
Sham
Tumor
c
Il6-KO
d
Cells
Live cells
Singlets
Leukocytes
SSC
FSC
Live-dead Aqua
FSC-A
CD45 - PerCP
Ly6C High Monos
Myeloid and Lymphoid
B cells
T cells
Neutrophils
Ly6C Low Monos
LCN2+ Neutrophils
CD11b - BB515
CD3 - PE-Cy7
CD19 - APC
Ly6C - APC-Cy7
Ly6G - BV421
Intracellular LCN2 - PE
Secondary only control
LCN2+ Leukocytes
e
Myeloid Cells
Lymphocytes
Relative Cell Abundance
LCN2 staining intensity 488-2

Supplementary Figure 2. Related to Figure 2. (A) Western blots used to quantify densitometry values for lung, liver, spleen, and bone marrow (n = 3-4 per group). Terminal plasma LCN2 levels in sham and tumor-bearing (B) WT and *Myd88-KO* mice (N = 5 per group for *Myd88*-KO sham, *Myd88*-KO tumor, and WT tumor groups; N = 4 for the WT sham group) and (C) WT and *IL6-KO* mice (N = 5 per group for *Il6-KO* sham, *Il6-KO* tumor, and WT tumor groups; N = 4 for the WT sham group). (D) Flow cytometry gating strategy for circulating immune cell populations and intracellular LCN2. (E) Quantification of peripheral myeloid and lymphoid cell subtypes (as a percentage of total myeloid or lymphoid cells) stained for intracellular LCN2; accompanying fluorescent intensity histograms for LCN2 staining intensity and relative cell abundance in sham and cachectic groups. Vertical dotted lines approximate gating threshold for positive intracellular LCN2 staining. Histograms represent combined overlays of individual mice (sham or tumor). For neutrophil histogram overlay, the sham control mice display minimal cell abundance, thus are marginally visible (n = 4 per group). A; LCN2 molecular weight = 23 kDa, GAPDH = 37 kDa. All data are expressed as mean ± SEM. B; blue = sham operation controls, red = KPC engrafted mice. E; Sham operation controls = grey/black, KPC = red.

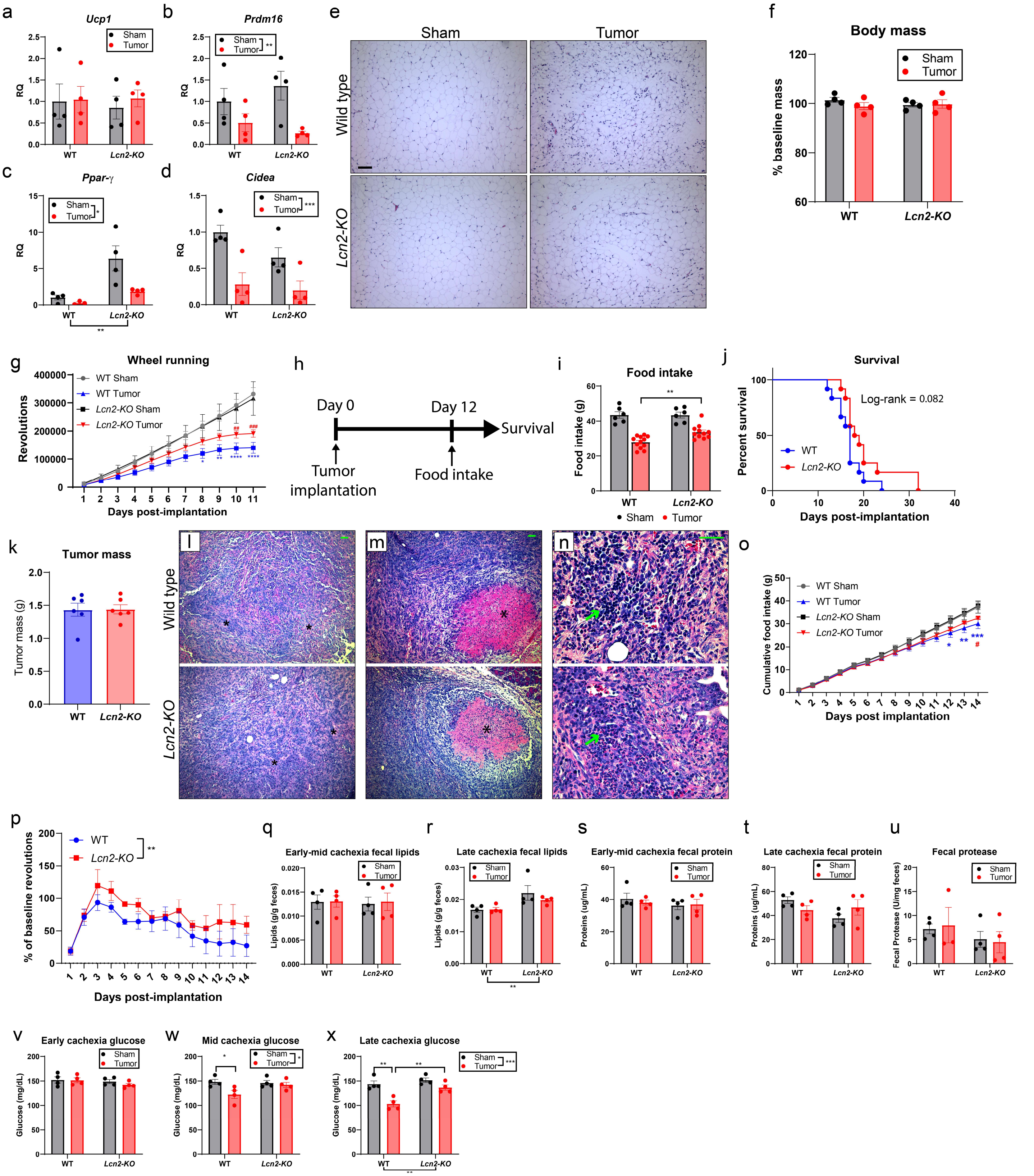

Supplementary Figure 3. Related to Figure 3. (A-D) Inguinal white adipose tissue gene expression analysis of browning genes *Ucp1, Prdm16, Ppar-γ,* and *Cidea* (N = 4 per group for all groups, except the WT tumor group in Figure C [N = 3]). (E) Representative H&E histochemical images of inguinal white adipose tissue of WT and *Lcn2-KO* mice after sham or tumor implantation (10x magnification, scale bar = 100 µm). (F) Terminal tumor-free mass of WT and *Lcn2-KO* mice after sham or tumor implantation (n = 4 per group). (G) Cumulative voluntary wheel running after tumor implantation or sham

operation in WT and *Lcn2-KO* mice (N = 6 per group for WT sham, WT tumor, and *Lcn2-KO* tumor groups; N = 7 for the *Lcn2-KO* sham group). (H) Experimental design for survival study and food consumption assessments. (I) Day 12 total food intake during survival study. (J) Kaplan Meier survival curve comparing WT and *Lcn2-KO* tumor-engrafted mice compared by log rank test. n = 6-12 per group for B-D. (K) Terminal tumor mass for WT and *Lcn2-KO* mice (n = 6 per group). Histopathologic characteristics of KPC allografts showing (L) ragged stromal infiltration, (M) geographical necrosis, and (N) acute inflammatory infiltrates after implantation into WT and *Lcn2-KO* mice (scale bar = 100 μm). (O) Cumulative food intake in WT and *Lcn2-KO* mice implanted with fewer KPC pancreatic cancer cells (0.75 x10^6) than in Figure 3 (n = 4 per group). (P) Normalized voluntary wheel running data in WT and *Lcn2-KO* mice orthotopically implanted with 0.75x10^6 KPC cells after a 3 week acclimation period (n = 4 per group). (Q) Early-mid and (R) late-stage cachexia fecal lipid levels in WT and *Lcn2-KO* mice (n = 4 per group). (S) Early-mid (days 1-6) and (T) late-stage (days 7-11) cachexia fecal protein levels in WT and *Lcn2-KO* mice (n = 4 per group). (U) Terminal fecal protease activity in WT and *Lcn2-KO* mice (n = 4 per group). (V) Early (day 4), (W) mid (day 8), and (X) late-stage (day 11) blood glucose levels in WT and *Lcn2-KO* mice (n = 4 per group). Repeated food intake and wheel running data were analyzed by a repeated-measures Two-way ANOVA followed by Bonferroni's post hoc test (G, O, P). Total food intake, gene expression data, body mass, fecal analyses, and glucose levels were analyzed by ordinary Two-way ANOVA followed by Bonferroni's post hoc test (A-D, F, I, Q-X). (J) Data was analyzed by a log-rank test (two-tailed). All data are expressed as mean ± SEM. A-F, I, Q-X; Sham operation controls = grey/black, KPC-engrafted mice = red. G, J, K, O, P; Gray = WT sham operation control; Black = *Lcn2-KO* sham operation control; Blue = WT KPC-engrafted mice; Red = *Lcn2-KO* KPC-engrafted mice.

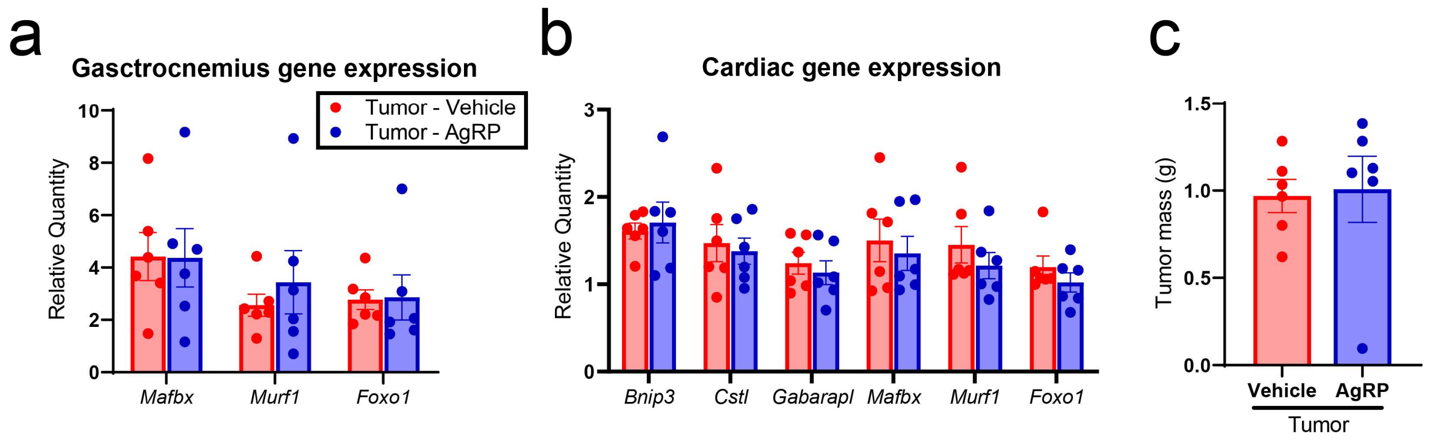

Supplementary Figure 4. Related to Figure 4. (A) Gastrocnemius and (B) cardiac tissue ubiquitin ligase and autophagy pathway gene analysis. (C) Terminal tumor mass. n = 6 per group. All data are expressed as mean ± SEM. Red = KPC-engrafted, ICV vehicle treated mice, Blue = KPC-engrafted, ICV AgRP treated mice.

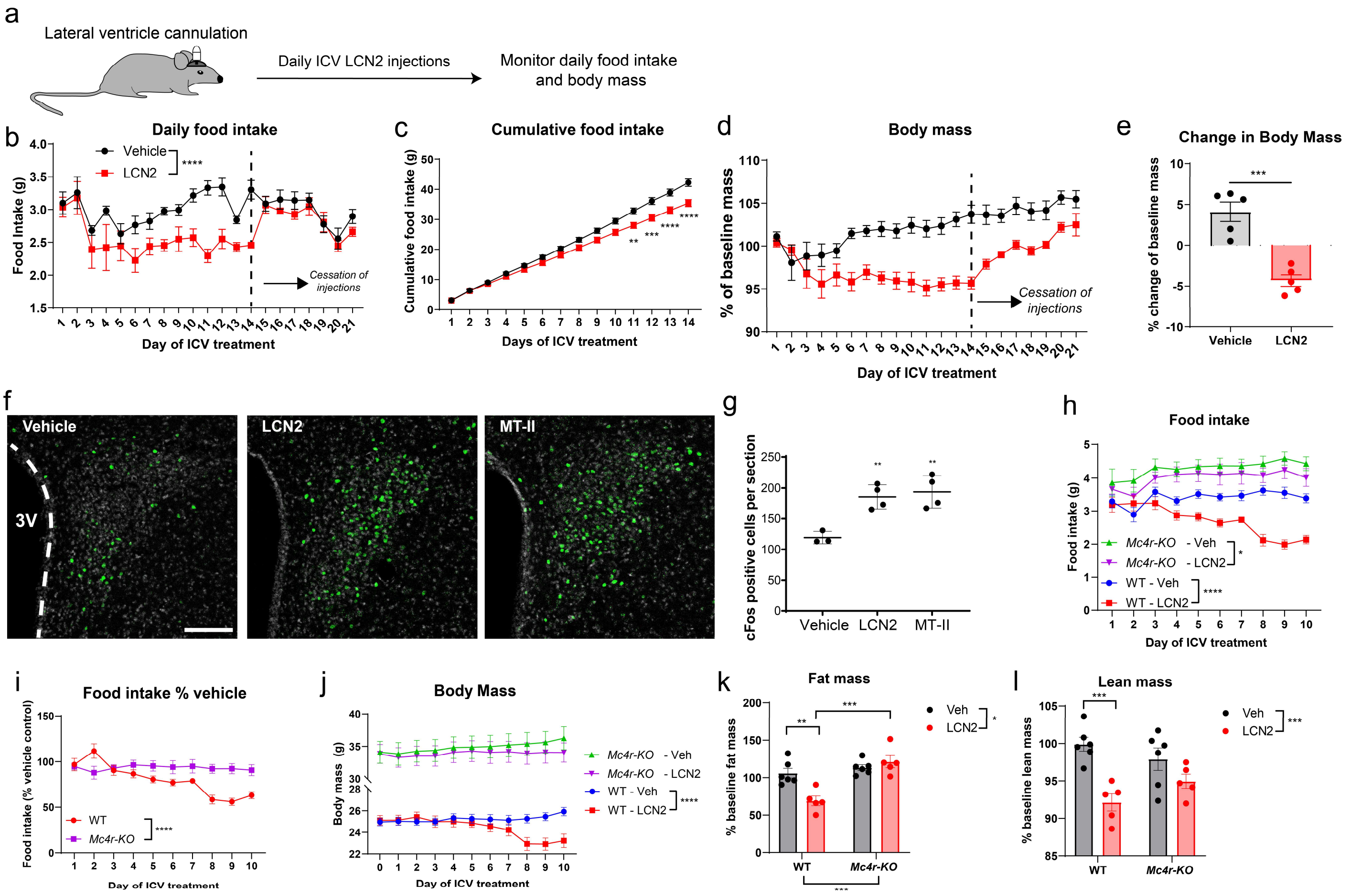

Supplementary Figure 5. Related to Figure 5. (A) Experimental protocol for ICV administration of LCN2 (40 ng) or vehicle control. (B) Daily and (C) cumulative food intake. (D) Daily change in body mass as a percentage of baseline mass. (E) Change in body mass at the end of the ICV treatment period (14 days). B-E; N = 5 per group. After day 14, ICV treatment was stopped and food intake and body weight data were collected for another week. (F) Representative images of paraventricular nucleus cFos staining 90 minutes after ICV administration of LCN2 (40 ng), MT-II (1 nmol), or vehicle (scale bar = 100 µm). (G) Quantification of cFos positive cells using the average count in three consecutive sections per mouse (N = 3 for the ICV vehicle group; N = 4 for the ICV LCN2 and MT-II groups). 3V = third ventricle. ICV = intracerebroventricular. (H) Daily food intake, (I) genotype-normalized food intake, (J) daily body mass, and terminal (K) fat and (L) lean mass in WT and *Mc4r-KO* mice receiving ICV vehicle or LCN2 (40 ng). H-L; N = 6 per group for WT ICV vehicle and Mc4rKO ICV vehicle groups; N = 5 per group for WT ICV LCN2 and Mc4rKO ICV LCN2 groups). All data are expressed as mean ± SEM. ICV = intracerebroventricular. A-E, K-L; Black = ICV Vehicle, Red = ICV LCN2. H-J; Blue = WT ICV vehicle injection, Red = WT ICV LCN2 injection, Green = Mc4r-KO ICV vehicle injection, Purple = Mc4r-KO ICV LCN2 injection.

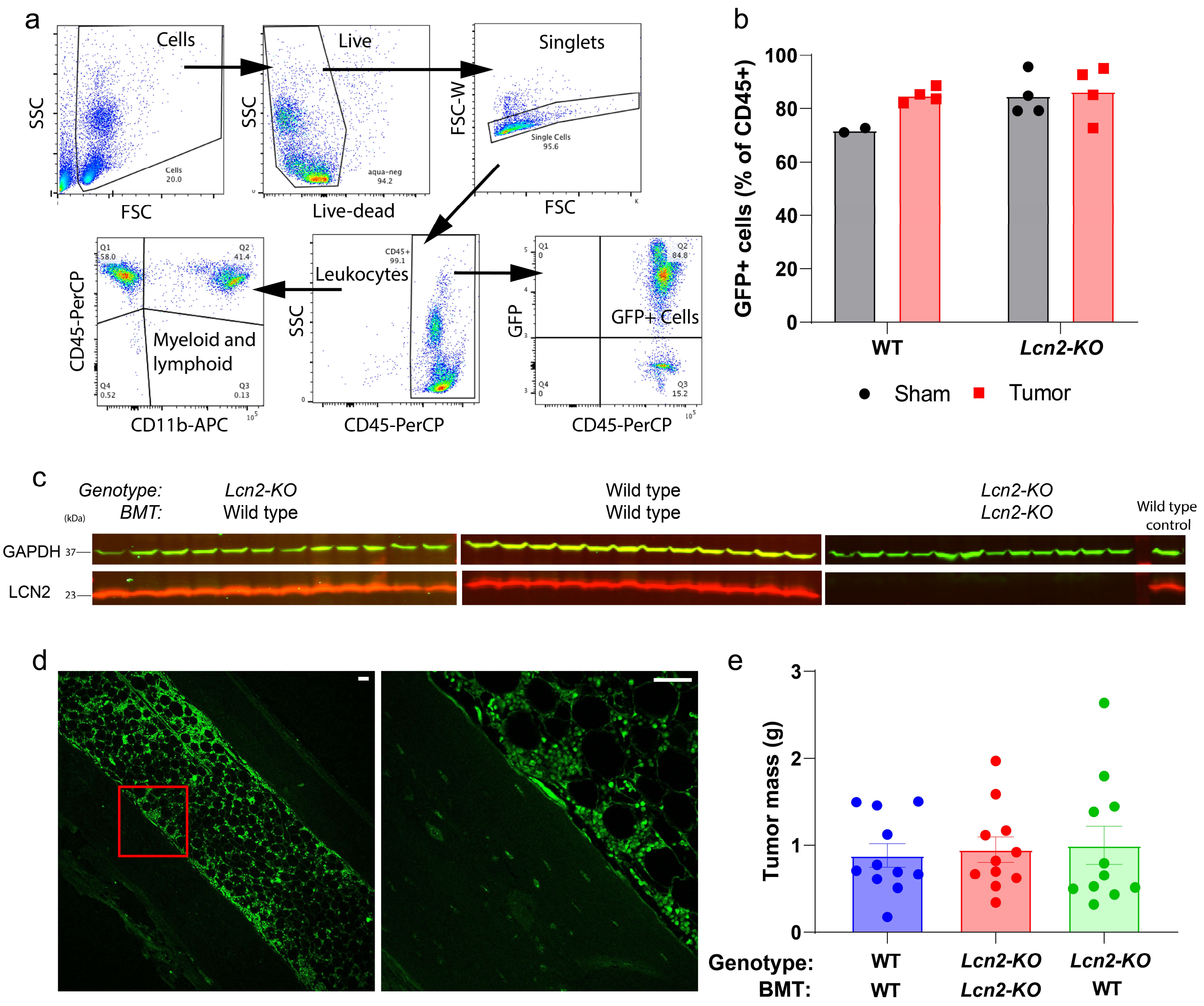

Supplementary Figure 6. Related to Figure 5. (A) Gating strategy for flow cytometry analysis of circulating GFP+ immune cells after generation of GFP+ bone marrow chimeras. (B) Quantification of GFP+ as a percentage of CD45+ cells in WT and *Lcn2-KO* mice after sham operation or tumor implantation. (C) Representative bone marrow Western blots confirming successful bone marrow engraftment and expression of LCN2. (D) Representative confocal images of cortical and spongy bone in a WT mouse after receiving Ly5.1 eGFP bone marrow transplantation (scale bar = 50μm). (E) Terminal tumor mass for all mice included in the bone marrow transplantation studies. C; LCN2 molecular weight = 23 kDa, GAPDH = 37 kDa. All data are expressed as mean ± SEM. BMT = bone marrow transplantation. For B, no statistics were performed, as only n = 2 was achieved for the WT sham operation control. E was analyzed by one-way ANOVA. B; Grey = sham operation control, Red = KPC-engrafted mice. E; Blue = WT/WT-BMT KPC-engrafted mice, Red = *Lcn2-KO*/*Lcn2-KO*-BMT KPC-engrafted mice, Green = *Lcn2-KO*/WT-BMT KPC-engrafted mice.

			Total (N = 100)
Age			62.3 (11.3)
Sex	Male		51
	Female		49
BMI (kg/m2)	Mean		27.2 (5.6)
	Binned	<18.5	4
		18.5-24.9	33
		25-29.9	39
		29.9-34.9	16
		>35	8
Days from diagnosis to treatment			32.6 (20.3)
Tumor location		Head	62
		Body	6
		Tail	4
		Overlapping	5
		NOS	23
Cancer Stage		0	2
		IA	0
		IB	4
		IIA	19
		IIB	41
		III	10
		IV	23
		Unknown	1
Neoadjuvant Therapy	Chemo	No	72
		Yes	12
		Unknown	16
	RT	No	81
		Yes	10
		Unknown	9
Tumor resection		No	33
		Yes	67
Adjuvant Chemotherapy		No	18
		Yes	49
Chemotherapy regimens		Gemcitabine	32
		FOLFIRINOX	14
		Gemcitabine/nab-paclitaxel	17
		FOLFIRINOX + Gemcitabine/nab-paclitaxel	2
		FOLFOX	1
		Gemcitabine/cisplatin	1
		Gemcitabine/HAP	4
		Unknown	11
Immune cell		NEUTROPHIL %	65.8 (16.6)
		LYMPHOCYTE %	19.3 (11.5)
		Neutrophil/Lymphocyte ratio	6.6 (7.7)
Plasma LCN2 (ng/mL)			150.3 (90.8)

CA19-9	890.8 (2838.3)

Supplementary table 1. Related to Figure 7A-C. Patient demographics, treatment, and biomarker data. Abbreviations: BMI, body mass index; NOS, not otherwise specified; HAP, hypoxic abdominal perfusion; FOLFIRINOX, 5-fluorouracil, leucovorin, irinotecan, and oxaliplatin; FOLFOX, 5-fluorouracil, leucovorin, and oxaliplatin. RT, radiotherapy; CA19-9, cancer antigen 19-9. Standard deviation presented in parentheses where appropriate.

			N = 22
Age			62.2 (12.9)
Sex	Male		13
	Female		9
BMI (kg/m2)	Mean		26.9 (4.8)
	Binned	<18.5	1
		18.5-24.9	6
		25-29.9	10
		29.9-34.9	5
Days from diagnosis to treatment			36.2 (23.8)
Tumor location		Head	12
		Body	2
		Tail	0
		Overlapping	0
		NOS	8
Cancer Stage		IA	1
		IB	0
		IIA	2
		IIB	5
		III	5
		IV	9
Neoadjuvant Therapy	Chemo	No	9
		Yes	4
		Unknown	9
	RT	No	16
		Yes	4
		Unknown	2
Tumor resection		No	15
		Yes	7
Adjuvant Chemotherapy		No	1
		Yes	6
Chemotherapy regimens		Gemcitabine	3
		FOLFIRINOX	5
		Gemcitabine/nab-paclitaxel	9
		FOLFIRIONX + Gemcitabine/nab-paclitaxel	1
		FOLFOX	1
		Unknown	2
Plasma LCN2 (ng/mL)		Baseline	217.5 (175.9)
		Follow up	263.5 (138.2)
CA19-9			1537.9 (4670.1)
Body Composition	SMI	Baseline	38.8 (8.1)
		Follow up	33.8 (13.8)
	Visceral adipose	Baseline	33.2 (27.9)
		Follow up	22.6 (38.6)

Supplementary table 2. Related to Figure 7E-F. Patient demographics, treatment, and biomarker data. Abbreviations: BMI, body mass index; NOS, not otherwise specified; FOLFIRINOX, 5-fluorouracil, leucovorin, irinotecan, and oxaliplatin; FOLFOX, 5-fluorouracil, leucovorin, and oxaliplatin. RT, radiotherapy; CA19-9, cancer antigen 19-9. Standard deviation presented in parentheses where appropriate.

			LCN2 <240 ng/mL (N = 111)	LCN2 >240 ng/mL (N = 17)	P value
Age			63.8 (10.9)	59.4 (13.9)	0.14
Sex	Male		56	7	0.480
	Female		55	10	
BMI (kg/m2)	Mean		27.3 (5.6)	27.8 (6.0)	0.73
	Binned	<18.5	4	0	0.930
		18.5-24.9	36	6	
		25-29.9	43	7	
		29.9-34.9	16	2	
		>35	10	2	
Days from diagnosis to treatment			34.1 (27.2)	31.9 (17.8)	0.75
Tumor location		Head	71	8	0.040
		Body	5	2	
		Tail	6	0	
		Overlapping	3	3	
		NOS	26	4	
Cancer Stage		0	2	0	0.590
		IA	1	0	
		IB	7	1	
		IIA	20	4	
		IIB	43	3	
		III	12	4	
		IV	26	5	
Median survival time (mo)			17.44	11	
Neoadjuvant Therapy	Chemo	No	82	11	0.670
		Yes	12	2	
		Unknown	17	4	
	RT	No	93	14	0.850
		Yes	9	2	
		Unknown	9	1	
Tumor resection		No	35	9	0.100
		Yes	76	8	
Adjuvant Chemotherapy		No	20	3	0.680
		Yes	56	5	
Chemotherapy regimens		Gemcitabine	36	3	0.110
		FOLFIRINOX	16	0	
		FOLFIRI	1	0	
		Gemcitabine/nab-paclitaxel	17	5	
		FOLFIRIONX + Gemcitabine/nab-paclitaxel	1	2	
		FOLFOX	1	0	
		Gemcitabine/cisplatin	2	1	
		Gemcitabine/HAP	3	1	
		Unknown	14	2	

Plasma LCN2 Diagnosis (ng/mL)	125.9 (46.8)	400.4 (136.5)	<0.01
CA19-9	929.6 (2845.4)	997.2 (2092.5)	0.93

Supplementary table 3. Related to Figure 7G. Patient demographics, treatment, and biomarker data dichotomized by a 240 ng/mL baseline LCN2 threshold. Abbreviations: BMI, body mass index; NOS, not otherwise specified; HAP, hypoxic abdominal perfusion; FOLFIRINOX, 5-fluorouracil, leucovorin, irinotecan, and oxaliplatin; FOLFOX, 5-fluorouracil, leucovorin, and oxaliplatin; FOLFIRI, 5-fluorouracil, leucovorin, and irinotecan. RT, radiotherapy; CA19-9, cancer antigen 19-9. Standard deviation presented in parentheses where appropriate. P-values calculated using Student's t-test for continuous variables, χ^2 or Fisher's Exact Test for categorical variables. When applicable, all statistical tests were two-tailed.

Target	Name	Manufacturer	Catalog	Dilution	FMO Prepared
CD11b BB515	Integrin alpha M	BioLegend	#101206	1:125	Yes
CD19 APC	Cluster of differentiation 19	BioLegend	#115541	1:125	No
CD3 PE-Cy7	Cluster of differentiation 3	BD Horizon	#564010	1:125	Yes
CD45 PerCP-Cy5.5	Protein tyrosine phosphatase, receptor type, C	BD Pharmingen	#552848	1:125	No
Ly6C APC-Cy7	Lymphocyte antigen 6 complex, locus C1	BioLegend	#128028	1:200	Yes
Ly6G BV421	Lymphocyte antigen 6 complex locus G6D	BioLegend	#127622	1:200	Yes
Live Dead Aqua	(N/A)	Invitrogen	#L34957	1:200	N/A
LCN2	Lipocalin 2	R&D	#AF1857	1:100	N/A
Anti-Goat-PE	F(ab')2-Donkey anti-Goat IgG (H+L) Cross-Adsorbed Secondary Antibody, PE	ThermoFisher Scientific	#31860	1:200	N/A

Supplementary table 4: Materials used for flow cytometry

Gene	Name	Catalog/sequence	Tissue(s)
Mafbx	Muscle atrophy F-box	Mm00399518_m1	Gastrocnemius, heart
Murf1	Muscle ring finger 1	Mm01185221_m1	Gastrocnemius, heart
Foxo1	Forkhead box O1	Mm00490672_m1	Gastrocnemius, heart
Bnip3	BCL2 Interacting Protein 3	Mm01275600_g1	Heart
Ctsl1	Cathespin L1	Mm00515597_m1	Heart
Gabarapl	GABA Type A Receptor Associated Protein Like	Mm00457880_m1	Heart
Il1b	Interleukin-1 beta	Mm01336189_m1	Hypothalamus, liver
Il1r1	Interleukin 1 receptor type 1	Mm00434237_m1	Hypothalamus
Il10	Interleukin 10	Mm01288386_m1	Hypothalamus
Nos2	Nitric oxide synthase 2	Mm00440502_m1	Hypothalamus
Apcs	Amyloid P component, serum	Mm00488099_g1	Liver
Il6	Interleukin 6	Mm00446190_m1	Liver
Orm1	Orosomucoid 1	Mm00435456_g1	Liver
Selp	P-selectin	Mm00441295_m1	Liver
18s	18s ribosomal subunit	4352930E	All
Ucp1	Thermogenin	Mm01244861_m1	Adipose tissue
Prdm16	PR domain containing 16	Mm00712556_m1	Adipose tissue
Ppar-gamma	Peroxisome proliferator-activated receptor gamma	Mm00440940_m1	Adipose tissue
Cidea	Cell Death Inducing DFFA Like Effector A	Mm00432554_m1	Adipose tissue

Supplementary table 5: Primer-probes used for qRT-PCR

Target (and conjugate)	Name	Manufacturer	Catalog	Dilution
GAPDH	Glyceraldehyde-3-Phosphate Dehydrogenase	Cell Signaling	#97166S	1:1000
LCN2	Lipocalin 2	R&D	#AF1857	1:800

Supplementary table 6: Antibodies used for western blotting
