# Supplementary material for: Lipocalin 2 mediates appetite suppression during pancreatic cancer cachexia
Source: Nat Commun. 2021 Apr 6;12:2057. doi: 10.1038/s41467-021-22361-3 (PMC8024334; doi:10.1038/s41467-021-22361-3)
Supplement: Supplementary file 3 — Reporting summary [file 41467_2021_22361_MOESM3_ESM.pdf]

nature research

Corresponding author(s): Daniel L Marks

Last updated by author(s): Mar 4, 2021

Reporting Summary

Nature Research wishes to improve the reproducibility of the work that we publish. This form provides structure for consistency and transparency in reporting. For further information on Nature Research policies, see our Editorial Policies and the Editorial Policy Checklist.

Statistics

For all statistical analyses, confirm that the following items are present in the figure legend, table legend, main text, or Methods section.

n/a	Confirmed	
☐	☒	The exact sample size (*n*) for each experimental group/condition, given as a discrete number and unit of measurement
☐	☒	A statement on whether measurements were taken from distinct samples or whether the same sample was measured repeatedly
☐	☒	The statistical test(s) used AND whether they are one- or two-sided<br>*Only common tests should be described solely by name; describe more complex techniques in the Methods section.*
☐	☒	A description of all covariates tested
☐	☒	A description of any assumptions or corrections, such as tests of normality and adjustment for multiple comparisons
☐	☒	A full description of the statistical parameters including central tendency (e.g. means) or other basic estimates (e.g. regression coefficient) AND variation (e.g. standard deviation) or associated estimates of uncertainty (e.g. confidence intervals)
☐	☒	For null hypothesis testing, the test statistic (e.g. *F*, *t*, *r*) with confidence intervals, effect sizes, degrees of freedom and *P* value noted<br>*Give P values as exact values whenever suitable.*
☒	☐	For Bayesian analysis, information on the choice of priors and Markov chain Monte Carlo settings
☒	☐	For hierarchical and complex designs, identification of the appropriate level for tests and full reporting of outcomes
☐	☒	Estimates of effect sizes (e.g. Cohen's *d*, Pearson's *r*), indicating how they were calculated

Our web collection on statistics for biologists contains articles on many of the points above.

Software and code

Policy information about availability of computer code

Data collection	Cross sectional muscle and fat area was collected using slice-o-matic and area measurements were exported to a deidentified excel sheet. CT images were saved in DICOM format using Osirix v11.0 (Pixmeo). The images underwent first-pass segmentation by an automated algorithm in MATLAB R2016a (MathWorks).
Data analysis	Determination of dichotomizing cutpoints of plasma LCN2 levels and survival outcomes utilized the Evaluate Cutpoints adaptive algorithm software in RStudio as described previously (Ogluszka et al., 2019). RNAseq: quality control checks were performed using the FastQC package and raw reads were normalized using the DESeq2 Bioconductor package. Kaplan Meier analysis was performed using IBM SPSS Statistics Suite (version 25). Flow cytometry data was analyzed using FloJo software and exported to excel files. All other analysis was performed using Prism 8.0 software.

For manuscripts utilizing custom algorithms or software that are central to the research but not yet described in published literature, software must be made available to editors and reviewers. We strongly encourage code deposition in a community repository (e.g. GitHub). See the Nature Research guidelines for submitting code & software for further information.

Data

Policy information about availability of data

All manuscripts must include a data availability statement. This statement should provide the following information, where applicable:

- Accession codes, unique identifiers, or web links for publicly available datasets
- A list of figures that have associated raw data
- A description of any restrictions on data availability

All data associated with this study are available in the main text, supplementary materials, or source data file. There are no restrictions on data availability. FCS flow cytometry files are available upon reasonable request. Sequencing data is available at GEO (GSE150061).

Field-specific reporting

Please select the one below that is the best fit for your research. If you are not sure, read the appropriate sections before making your selection.

[x] Life sciences [ ] Behavioural & social sciences [ ] Ecological, evolutionary & environmental sciences

For a reference copy of the document with all sections, see nature.com/documents/nr-reporting-summary-flat.pdf

Life sciences study design

All studies must disclose on these points even when the disclosure is negative.

Sample size	Sample size was determined based on prior experience with these pancreatic cancer models of cachexia studies, such that we would readily distinguish behavioral and molecular outcomes between sham-operation and tumor-bearing animals (Michaelis et al., 2017). Specifically, our group previously determined that an N >/= 4 readily distinguishes the cachexia parameters studied herein, including behavior (food intake and locomotion) and gene expression in cachexia-sensitive tissues. For human studies, data were collected in a retrospective manner from a preexisting database of pancreatic cancer patients. As such, no power calculations were performed, but patients were selected if their data included basic demongraphics/tumor characteristics, as well as an initial blood draw. Subsequent analyses, including survival and body composition, then required subset selection of this cohort to include survival data and axial CT scan body composition analyses, respectively.
Data exclusions	Our a priori policy was to exclude animals and their samples from analysis (1) if tumor implantation was unsuccessful, or (2) if random events over the course of the study with confounding potential would put the animal in a biologically distinct category unrelated to their tumor status or treatment allocation (such as treatment administration complications resulting in large volume bleeding, a cage disruption such as water leaking, etc.).
Replication	To ensure the predominant effect of improve food intake in the setting of LCN2KO, studies comparing LCN2KO and WT animals were replicated at least three times, including cachexia measures (food consumption, wheel running, etc.). All attempts at replication were successful for this study. Similarly, the behavioral findings reported in Figure 1A, and Figure S5B were replicated independently.
Randomization	Randomization of groups was performed to ensure variables related to cachexia were balanced across tumor and sham-operated animals, using the covariates initial body weight, food intake, and wheel running (where appropriate). Animals were further randomized for these covariates when multiple treatment groups were present, such that cachexia-related parameters were expected to be evenly distributed between groups. T-tests (for two groups) or ANOVA (for 3+ groups) were performed within studies for all relevant variables to ensure no significant differences were present at baseline for any of the aforementioned characteristics, such that changes at endpoint could be attributed to the disease state or treatment group rather than baseline differences. Concerning experiments that were not performed with tumor implantation, mice were randomized by covariates of initial body weight and food intake.
Blinding	Blinding was performed for any qualitative analysis. In animal models, investigators were aware of which animals were tumor-bearing or sham-operated, but blinded to genotype. However, all collected data were quantitative in nature during this phase. Gene expression studies and samples acquired from these studies were coded and blinded for downstream gene expression analyses.

Reporting for specific materials, systems and methods

We require information from authors about some types of materials, experimental systems and methods used in many studies. Here, indicate whether each material, system or method listed is relevant to your study. If you are not sure if a list item applies to your research, read the appropriate section before selecting a response.

Materials & experimental systems

n/a	Involved in the study	
[ ]	[x]	Antibodies
[ ]	[x]	Eukaryotic cell lines
[x]	[ ]	Palaeontology and archaeology
[ ]	[x]	Animals and other organisms
[ ]	[x]	Human research participants
[x]	[ ]	Clinical data
[x]	[ ]	Dual use research of concern

Methods

n/a	Involved in the study	
[x]	[ ]	ChIP-seq
[ ]	[x]	Flow cytometry
[x]	[ ]	MRI-based neuroimaging

Antibodies

Antibodies used

Antibodies and corresponding manufacturers/catalog numbers are described in the Supplementary tables 4 and 6.
Flow cytometry:
Target Name Manufacturer Catalog Dilution FMO Prepared
CD11b BB515 Integrin alpha M BioLegend #101206 1:125 Yes
CD19 APC Cluster of differentiation 19 BioLegend #115541 1:125 No

CD3 PE-Cy7 Cluster of differentiation 3 BD Horizon #564010 1:1
25 Yes
CD45 PerCP-Cy5.5 Protein tyrosine phosphatase, receptor type, C BD Pharmingen #552848 1:125 No
Ly6C APC-Cy7 Lymphocyte antigen 6 complex, locus C1 BioLegend #128028 1:200 Yes
Ly6G BV421 Lymphocyte antigen 6 complex locus G6D BioLegend #127622 1:200 Yes
Live Dead Aqua (N/A) Invitrogen #L34957 1:200 N/A
LCN2 Lipocalin 2 R&D #AF1857 1:100 N/A
Anti-Goat-PE F(ab')2-Donkey anti-Goat IgG (H+L) Cross-Adsorbed Secondary Antibody, PE ThermoFisher Scientific #31860 1:200 N/A
Target (and conjugate) Name Manufacturer Catalog Dilution

Western Blot:
GAPDH Glyceraldehyde-3-Phosphate Dehydrogenase Cell Signaling #97166S 1:1000
LCN2 Lipocalin 2 R&D #AF1857 1:800

Validation

LCN2 antibodies utilized in this study were validated for antigen specificity using Western blot, immunohistochemistry, and flow cytometry. In the manuscript, we evidence this validation through absent staining in Lcn2-KO mouse tissue, including the bone marrow (Figure S2A) Flow cytometry antibodies were validated in previous studies (Burfeind et al., 2020) and by the manufacturers. Specific details for manufacturer validations are present on their websites, but are not elaborated here due to the large number of antibodies used in this study.

Eukaryotic cell lines

Policy information about cell lines

Cell line source(s)

We thank Drs. Elizabeth Jaffee (KPC), David Tuveson (FC1242, FC1199, and FC1245), and Robert Vonderheide (4662) for graciously providing the syngeneic pancreatic cancer cell lines used for our studies.

Authentication

We have not personally authenticated the cell lines in these studies using a professional service; however, they form tumors consistent with moderately-to-poorly differentiated pancreatic ductal adenocarcinoma in vivo.

Mycoplasma contamination

All cell lines in this study undergo routine mycoplasma testing, and have been consistently negative. They additionally have undergone PCR screening by IDEXX Bioresearch for 40 microbial and viral contaminants, for which they were negative.

Commonly misidentified lines
(See ICLAC register)

We did not use any commonly misidentified lines in this paper.

Animals and other organisms

Policy information about studies involving animals; ARRIVE guidelines recommended for reporting animal research

Laboratory animals

C57BL6/J mice (JAX, cat. #000664), LCN2KO mice (JAX, cat. #024630), Melanocortin 4 receptor knockout (Mc4r-KO, JAX catalog number 032518), Interleukin 6 knockout (Il-6-KO, JAX catalog number 002650), and Myeloid differentiation primary response gene 88 knockout (MyD88-KO, JAX catalog number 009088) were used in these studies, with implantation occurring at 7-10 weeks of age. All mice were male.

Wild animals

No wild animals were used in this study.

Field-collected samples

No field collected samples were used in this study.

Ethics oversight

Animal studies were performed in accordance with the Oregon Health and Science University IACUC, with principles following the NIH Guide for the Care and Use of Laboratory Animals.

Note that full information on the approval of the study protocol must also be provided in the manuscript.

Human research participants

Policy information about studies involving human research participants

Population characteristics

Patients diagnosed with pancreatic cancer or age-matched healthy controls at Oregon Health & Science University between 2012-2017.

Recruitment

Patients were recruited by the Oregon Clinical and Translational Research Institute and Brenden Colson Center for Pancreatic Care. Associated patient information and tissues are stored by the respective organizations at Oregon Health & Science University. All patient data and samples were anonymized.

Plasma samples from men and women age 40-82 years old diagnosed with pancreatic cancer were procured through the Brenden Colson Center for Pancreatic Care (Portland, Oregon) through the Oregon Pancreatic Tumor Registry (OPTR). These samples were collected at the time of diagnosis, and for some patients, in follow-up visits. Age- and sex-matched control samples from patients with no clinical evidence of disease were procured from the Oregon Clinical and Translational

	Research Institute. Specifically, patients that were seen at OHSU with a similar clinical work-up as the pancreatic cancer group, but deemed to have no evidence of pancreatic disease, were included in this study. Informed consent was obtained by these respective organizations for all samples and data utilized in this study. Blood was drawn through venipuncture and plasma and the buffy coat were separated. Samples were then stored at -80°C until assayed. Given the retrospective and anonymized nature of the human samples and data reported in the manuscript, these studies were deemed non-humans research, and IRB approval was waived by the OHSU IRB.
Ethics oversight	Given the retrospective and anonymized nature of the human samples and data reported in the manuscript, these studies were deemed non-humans research, and IRB approval was waived by the OHSU IRB.

Note that full information on the approval of the study protocol must also be provided in the manuscript.

Flow Cytometry

Plots

Confirm that:

☒ The axis labels state the marker and fluorochrome used (e.g. CD4-FITC).

☒ The axis scales are clearly visible. Include numbers along axes only for bottom left plot of group (a 'group' is an analysis of identical markers).

☒ All plots are contour plots with outliers or pseudocolor plots.

☒ A numerical value for number of cells or percentage (with statistics) is provided.

Methodology

Sample preparation	Mice were anesthetized using a ketamine/xylazine/acepromazine cocktail and whole blood was collected by cardiopuncture. 200 µL of whole blood was then treated with 1x RBC lysis buffer (Invitrogen) for 10 minutes then incubated in 100 µL of PBS containing antibodies for 45 minutes at 4°C. For intracellular LCN2 staining, cells were then fixed and permeabilized for 10 minutes, washed with 1x permeabilization buffer (Invitrogen), and stained with primary antibody for 45 minutes at 4°C. Cells were stained with a secondary antibody for 1 hour at 4°C and resuspended in 200 µL of RPMI +5% FBS for analysis. Cells were thoroughly washed between steps with either RPMI +5% FBS (prior to fixation/permeabilization) or 1x permeabilization buffer (after fixation/permeabilization).
Instrument	BD LSR II
Software	Flow cytometry data was analyzed using FlowJo software.
Cell population abundance	Each analyzed cell population abundance is quantified in the main text (Figures 2, 3) or supplemental (Figure S2) figures. These data are not based on sorted populations, and the % of positive cells was determined after gating on subset specific markers.
Gating strategy	Described in Figures S2. Detailed gating strategy is provided in the methods section: Cells were gated on LD, SSC, and FSC singlet. Immune cells were defined as CD45+ cells. Myeloid cells were defined as CD45highCD11b+, and lymphocytes were defined as CD45highCD11b-. Myeloid cells were further gated into Ly6Clow monocytes (Ly6ClowLy6G-), Ly6Chigh monocytes (Ly6ChighLy6G-), and neutrophils (Ly6CmidLy6G+). Lymphocytes were grouped as either CD3+ T-cells or CD19+ B-cells. The resulting T-cell gate was further stratified into CD4+ or CD8+ T-cells. All of the aforementioned groups were intracellularly stained for LCN2. Flow cytometry analysis was performed on the LSRII analytic flow cytometer.

☒ Tick this box to confirm that a figure exemplifying the gating strategy is provided in the Supplementary Information.
